# Trends in Family Planning in Russia, 1994–2003

**DOI:** 10.1363/4104009

**Published:** 2009-03

**Authors:** Francesca Perlman, Martin McKee

**Affiliations:** Francesca Perlman is clinical lecturer and Wellcome Intermediate Clinical Fellow Martin McKee is professor of European public health—both at the European Centre on Health of Societies in Transition, London School of Hygiene and Tropical Medicine

## Abstract

**CONTEXT:** Although Russian women have adequate knowledge of modern contraceptives, their level of use of these methods has been low, and abortion rates remain relatively high.

**METHODS:** In 1994–2003, sexually active women aged 18–49 were interviewed about their contraceptive use as part of the Russia Longitudinal Monitoring Survey. Trends in contraceptive use were examined. Multivariate analyses were conducted to identify characteristics associated with reliable contraceptive use (IUD use or consistent oral contraceptive use) in 1994 and 2003.

**RESULTS:** In each year, about 25% of sexually active women had used no contraceptive method in the past month, and 20% had used traditional methods. Prevalence of barrier method use increased from 9% to 21% between 1994 and 2003, while that of IUD use declined from 34% to 21%. These changes were especially pronounced in Moscow and St. Petersburg, and among younger women. Common reasons for nonuse were irregular sexual relations (cited by 29% of nonusers in 2003), desire for pregnancy (22%), perceived inability to get pregnant (15%), feeling that contraceptives are uncomfortable or unpleasant (15%), health problems (11%) and the availability of abortion (6%). In 1994 and 2003, the odds of reliable contraceptive use were elevated among women with at least a secondary education (odds ratios, 1.5–1.7), and were reduced among smokers (0.6–0.7).

**CONCLUSIONS:** Modern, effective contraceptive use has not increased among sexually active Russian women. Growing use of barrier contraceptives may reflect HIV awareness. Obstacles to effective contraceptive use, such as attitudes and health service factors, need further clarification.

Internationally, high abortion rates often are considered an indication that women's access to effective contraceptives is inadequate.^1^ In Russia, rates of induced abortion have long been high,^2^–^4^ and abortion-related complications are common;^5^–^7^ the limited information available suggests that use of modern family planning methods varies widely by region but is generally low.^8^ Recent political concern about declining fertility, and consequently population size,^9^ has resulted in government pressure on family planning organizations to limit the provision of family planning^5^,^8^,^10^ and in legislation to restrict abortion.^8^ Although these policy proposals are founded on reasonable knowledge of the number of abortions, relatively little is known about contraceptive use in Russia. In this study, we seek to understand how patterns of contraceptive use have changed on a national level and how these changes relate to national trends in abortion and fertility.

## FAMILY PLANNING IN THE SOVIET ERA

Historically, induced abortion was the most commonly used method of preventing unwanted childbirth in the Soviet Union. Abortion was legalized by the Bolshevik regime in 1920; this legislation was revoked during the 1930s,^11^ and then reinstated in the 1950s. By the 1960s, the abortion rate was 160 per 1,000 women aged 15–49;^5^ this high rate continued until the 1980s, when abortion levels began to decline,^5^ particularly in Estonia and Latvia.^12^ Nonetheless, in 1988 the Soviet Union accounted for 10–20% of the world's abortions.^2^ Official data indicate that women aged 15–49 had 1.2 abortions for every live birth; in some regions, they had seven abortions for every live birth, the highest ratio ever recorded.^2^ Moreover, these figures may be underestimates: The true ratios may have been twice as high if illegal abortions are taken into account.^2^ In a survey conducted in St. Petersburg shortly after the collapse of the Soviet Union, only 14% of women aged 15–65 reported that they had never had an abortion. On average, respondents had had three abortions by age 25, and a small proportion had had more than 20 in their lifetime.^13^

Lack of access to a wide range of contraceptives, coupled with limited knowledge and information, was thought to underlie these high abortion rates.^2^ The state had a monopoly on medical supplies, modern contraceptives were in short supply^14^ and the quality of Soviet condoms was notoriously poor.^15^ Misinformation may have influenced attitudes toward contraception: The introduction of oral contraceptives in the 1970s was accompanied by government propaganda warning women and doctors of many unsubstantiated risks, and the officially published contraindications applied to 80–90% of potential users.^14^ The government's stance may have reflected its concerns about ensuring regular supplies to a widely dispersed population.^16^

Nevertheless, use of modern contraceptives increased gradually during the 1980s,^5^ although use remained much less frequent than it was in the West. In each of five studies conducted during that period (three in Moscow, one in Saratov and Moscow, and one in Tartu and Moscow), just 2–3% of women were using oral contraceptives, and approximately 10% were using IUDs,^14^ which were often inserted following abortion.*^3^ However, more than half of women in these studies were using traditional methods, such as douching or withdrawal, which are unreliable,^17^ and many sexually active women were taking no precautions to avoid unwanted pregnancy.^14^

## RECENT TRENDS

Access to contraceptives appears to have improved in Rassia following the disintegration of the Soviet Union. Between 1989 and 1993, the proportion of St. Petersburg women who believed that access was “adequate” rose from 12% to 38%; nonetheless, even in 1991, 41% of women reported using traditional methods.^13^ Women younger than 25 were more likely than older women to be using a modern method; 35% were using an IUD, and 10% oral contraceptives.^13^ A 1996 survey in two cities (Perm and Yekaterinberg) and one rural area (Ivanovo Province) showed that one-third of women aged 15–44 were using an IUD; however, only one in 10 were using condoms, and fewer still oral contraceptives.^18^ Another option became available when sterilization was legalized in 1993, overturning a ban that had been in effect since the 1930s.^5^

Despite these advances, the availability of family planning was limited during the early 1990s; only 1% of 15–67-year-old women in St. Petersburg believed that services were satisfactory, and most resented paying the necessary bribes.^13^ The low level of clinician knowledge was a further obstacle. In one study, just half of gynecologists reported having received training in family planning, and fewer than two-thirds knew how oral contraceptives worked.^19^ However, women's awareness of modern contraceptive methods was improving, and was almost universal in Yekaterinberg and Perm by 1996.^18^

Between 1990 and 2000, the number of abortions in Russia declined by half.^3^ Although this decrease coincided with a major reduction in fertility, the ratio of abortions to live births declined only slightly in the early and mid-1990s, from 2.04 abortions per live birth in 1990 to 1.92 in 1996; this was followed by a steeper decline, to 1.56 abortions per live birth in 2000.^6^ While changes in the recording of abortions occurred after the breakup of the Soviet Union, the decline in the abortion rate is thought to be genuine, since it was documented in both survey and official data.^3^ The reliability of the survey data is further supported by evidence that women almost always report a “miniabortion” (vacuum aspiration during the first seven weeks of pregnancy) as an abortion,^20^ although they do so less often if they have had multiple abortions.^21^

Nonetheless, abortion remains much more common in Russia than in western Europe.^8^ Moreover, Russia's total fertility rate has fallen sharply, from 2.0 births per woman in 1989 to 1.2 in 1999; despite a subsequent rise, it was still only 1.3 in 2004.^22^ The combination of low fertility and high premature adult mortality has led to a shrinking population.^9^ However, it is uncertain whether improved access to contraceptives accounts for the declines in abortion and fertility. Some researchers have argued that fertility has declined because the pronatalist policies of the 1980s led women to reduce the intervals between births, so that by the 1990s many women had achieved their desired family size;^23^ however, this theory does not explain why the fertility rate remains low today.

At least two structural and political factors in Russia may be promoting abortion and limiting access to effective contraceptives. First, health care providers may have little financial incentive to provide family planning services. During the Soviet era, a three-day hospital stay was mandatory for an abortion, and even now the procedure (excluding miniabortions) must take place in a hospital; as a result, abortions represent an important source of income for providers and help justify the retention of otherwise underutilized hospital facilities.^5^ Second, national concern about declining fertility has led to policies that may have detrimental effects on family planning. For example, government financial incentives encourage women to have more children,^9^ legislation enacted in 2003 reduced the number of indications for legal abortion,^8^ and the government has expressed little support for—and sometimes actual opposition to—family planning programs.^5^,^10^

In the present study, we used data from 1994–2003 to investigate trends in contraceptive use, correlates of these trends and the reasons that women give for nonuse. We hypothesized that the proportion of women who used contraceptives regularly had increased; that use of reliable modern methods of contraception had increased, and use of traditional methods had declined commensurately; and that use of modern methods had diffused from a select group of women to the wider female population, such that social and demographic differences between users and nonusers had declined.

## METHODS

### Sampling Procedure

We used data from eight rounds (1994–2003) of Phase2 of the Russia Longitudinal Monitoring Survey (RLMS), a panel study of households and the individuals within them.† Participants came from 38 population centers across the Russian Federation. St. Petersburg and Moscow were intentionally included, and the remaining 36 districts, or primary sampling units, were sampled by stratifying districts according to socioeconomic criteria, and selecting from each stratum with probability proportional to size. Within the primary sampling units, urban and rural secondary sampling units (census enumeration districts and villages, respectively) were selected, from each of which 10 households were chosen from lists developed by the investigators. The first dwelling was selected randomly, and the remainder at regular intervals. All available household members participated in the survey. Thus, the sampling procedure was designed to provide a study population that was broadly representative of the national population, but that also included the two principal cities. In each study round, newly recruited households replaced ones that left. The turnover between rounds was 10–20%.^24^ Further details about the study methods may be found on the RLMS Web site.^25^

### Variables

#### • Contraceptive use

Female survey respondents were asked, “Have you used birth control in the last 30 days?” If they had, they were asked which of the following was their main method: douching, counting the fertile days of the menstrual cycle, withdrawal, condoms, oral contraceptives, IUD, implant, injectable, diaphragm, spermicide (lotions, suppositories, foam, jelly), sterilization (male or female), or other. We collapsed these methods into seven categories: traditional methods (douching, counting days, withdrawal), barrier methods (condoms, diaphragm, spermicide), hormonal methods (oral contraceptives, injectable, implant), IUD, sterilization, other methods and no method.

Women who were not using birth control were asked why this was so. Possible answers were that the woman wanted to get pregnant; was physically unable to get pregnant; had health problems; was unable to obtain a method; could not afford a method; felt that birth control was uncomfortable or unpleasant to use; had sex infrequently; had no husband or partner; or knew that as a last resort, she could have an abortion to end an unwanted pregnancy. Women who said they had used a method in the past 30 days also were asked if they had failed to use a method at any time during that period; those who replied yes were asked to give a reason from the same list.

#### • Other

We categorized women into four age-groups: 18–24, 25–34, 35–44 and 45–49. Place of residence was classified as metropolitan (Moscow and St. Petersburg) or other, and marital status as married or cohabiting, single, divorced or widowed. Educational attainment was categorized as less than secondary, completed secondary or higher. Household wealth was measured on the basis of whether the household had a color television, video recorder, car, washing machine and dacha; an asset score of 0-5 was assigned.

Women were asked whether they were current smokers, and about the frequency and quantity of their alcohol consumption, which was categorized as moderate (if they reported consuming less than 80 g of pure alcohol per occasion), binge (if they reported 80 g or more) or no alcohol consumption in the past month.

Finally, the survey included three reproductive measures: whether the woman had ever had a live birth, whether she had had an abortion in the past year and whether she wanted to have a (or another) child.

### Analyses

Our analyses focused on women aged 18–49 who reported having had sex in the past 30 days, and excluded those who were not using contraceptives because they had no husband or sexual partner. Women who believed they were infertile or who were trying to conceive were excluded from the analyses of contraceptive use. Data from each year were analyzed separately.

We calculated the prevalence of use for the seven contraceptive categories in each study round, standardizing the prevalence for each year to the value that would be expected if respondents' ages (in five-year age-groups) matched those of the 1994 study population. We repeated these analyses separately by age-group and by place of residence. We then summarized this information according to reliability of contraceptive method: We classified the IUD and hormonal contraceptives used regularly as the most reliable; barrier methods used regularly as the next most reliable; and any of the above methods used irregularly or any other method as unreliable. We also examined the reasons women gave for nonuse or irregular use of contraceptives, and we assessed the proportion of barrier and hormonal contraceptive users who did not use their method on some occasions.

Finally, for the earliest and latest study years (1994 and 2003), we performed multivariate analyses of the characteristics associated with use of the most reliable contraceptives. For each year, we created two models. The first was adjusted for age and marital status, and the second for those two variables as well as education, residence, smoking, alcohol consumption, household wealth and whether women had had a live birth. Analyses were adjusted for clustering by survey site (including census district).

## RESULTS

In each round of the survey, at least 2,500 women aged 18–49 (approximately 80% of women in this age-group) were sexually active. The mean age of these respondents was 33-34, and was similar in metropolitan and nonmetropolitan areas (Table 1, page 42). In 1994, 12% of the women were residents of Moscow or St. Petersburg; this proportion gradually declined to 5% by 2000, and subsequently increased after additional recruitment in the two cities was initiated in 2001.

**TABLE 1:** Selected characteristics of sexually active women aged 18–49, Russia Longitudinal Monitoring Survey, 1994–2003

Characteristic	1994 (N=2,722)	1995 (N=2,601)	1996 (N=2,576)	1998 (N=2,641)	2000 (N=2,790)	2001 (N=3,167)	2002 (N=3,245)	2003 (N=3,304)
MEANS								
**Age**								
All	33.5	33.9	33.7	33.7	33.4	33.3	33.3	33.1
Moscow/St. Petersburg	33.8	34.7	34.1	34.6	34.9	33.7	34.0	33.9
Other	33.4	33.8	33.7	33.7	33.3	33.2	33.2	33.0
% DISTRIBUTIONS								
**Age**								
18–24	20.8	20.6	22.3	22.3	23.8	24.3	24.1	24.2
25–34	31.8	28.9	28.6	27.7	28.8	29.6	30.7	31.8
35–44	34.0	35.0	34.3	34.2	31.9	29.9	28.7	27.4
45–49	13.5	15.5	14.8	15.6	15.5	16.1	16.6	16.7
**Residence**								
Moscow/St. Petersburg	11.7	9.8	8.5	6.6	5.2	13.8	14.5	12.8
Other	88.3	90.2	91.5	93.4	94.8	86.2	85.5	87.2
Total	100.0	100.0	100.0	100.0	100.0	100.0	100.0	100.0

*Notes:* Women were considered sexually active if they reported having had sex in the past 30 days. Women were excluded if they believed they were infertile or they were trying to conceive. Percentages may not total 100.0 because of rounding.

**FIGURE 1:**
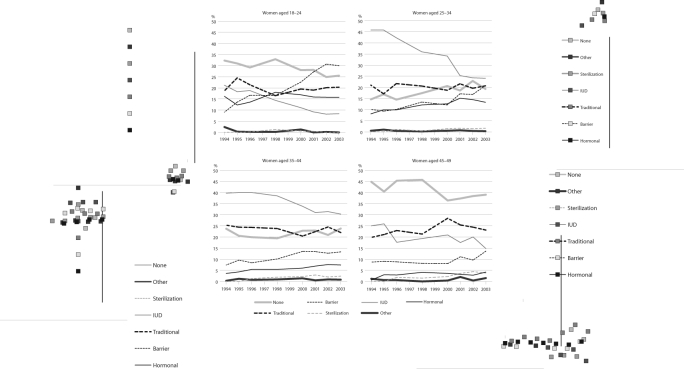
Percentage of sexually active women aged 18–49 who were using selected contraceptive methods, by age-group and year *Notes*: Women were considered sexually active if they reported having had sex in the past 30 days. Women were excluded if they believed they were infertile or they were trying to conceive. Surveys were not conducted in 1997 and 1999.

Between 1994 and 2003, the proportion of sexually active women who reported not using contraceptives was consistently about 25% (Table 2, page 43). However, changes are apparent in the types of contraceptives that women used. The prevalence of barrier method use increased from 9% to 21% of sexually active women, while prevalence of IUD use declined by a similar margin, from 34% to 21%. Among 18–24-year-olds, the prevalence of barrier method use increased from 9% to 30%, while that of IUD use declined from 21% to 8% (Figure 1). The decline in prevalence of IUD use was even greater among women aged 25–34 (from 45% to 25%), while it was smaller among women aged 35–44 (from 40% to 30%) and those aged 45–49 (from 25% to 14%). The prevalence of hormonal contraceptive use increased during the study period from 7% to 11% (Table 2); more than 95% of women reporting these methods were oral contraceptive users (not shown). The prevalence of hormonal contraceptive use was highest among 18–24-year-olds and was stable in that age-group (16% in both 1994 and 2003); it was lower but rose slightly among other age-groups. Traditional methods were used by about 20% of respondents throughout the study, regardless of age-groups. Dual method use was rare: In 2003, only 4% of respondents who reported barrier method use were also using oral contraceptives or IUDs (not shown).

**TABLE 2:** Percentage distribution of sexually active women aged 18–49, by contraceptive method used, according to place of residence and year

Residence/method	1994	1995	1996	1998	2000	2001	2002	2003
**All**								
None	26.0 (23.6–28.3)	24.6 (21.9–27.4)	23.8 (21.3–26.3)	25.9 (23.5–28.2)	26.1 (23.8–28.5)	25.6 (23.5–27.7)	24.7 (22.7–26.7)	24.7 (23.3–26.2)
Barrier**	9.0 (7.4–10.5)	10.7 (8.7–12.8)	11.1 (9.2–13.1)	12.8 (11.0–14.6)	14.6 (12.7–16.5)	18.1 (16.2–20.0)	18.6 (16.7–20.4)	20.8 (19.4–22.2)
IUD***	34.3 (32.1–36.5)	33.8 (31.4–36.1)	32.4 (30.0–34.9)	29.1 (27.0–31.2)	26.0 (24.0–28.0)	21.6 (19.9–23.3)	21.7 (20.0–23.4)	20.5 (19.4–21.7)
Hormonal**	7.4 (5.9–8.9)	8.8 (7.0–10.7)	8.6 (7.1–10.2)	10.1 (8.4–11.8)	10.1 (8.5–11.7)	10.9 (9.5–12.4)	11.2 (9.8–12.7)	10.6 (9.6–11.6)
Traditional	22.4 (20.2–24.5)	21.3 (18.7–23.8)	22.5 (20.2–24.9)	20.5 (18.5–22.5)	20.3 (18.2–22.4)	21.3 (19.4–23.2)	21.7 (19.8–23.6)	21.2 (19.8–22.5)
Sterilization***	0.0 (0.0–0.0)	0.0 (0.0–0.0)	1.0 (0.5–1.5)	1.2 (0.6–1.7)	1.5 (1.0–2.1)	2.0 (1.4–2.6)	1.6 (1.0–2.1)	1.7 (1.3–2.0)
Other	1.1 (0.3–1.8)	0.8 (0.4–1.2)	0.4 (0.1–0.8)	0.4 (0.1–0.7)	1.3 (0.5–2.0)	0.6 (0.3–0.9)	0.5 (0.2–0.8)	0.5 (0.3–0.7)
**Moscow/St. Petersburg**								
None	24.9 (18.5–31.3)	24.9 (15.7–34.2)	24.4 (17.0–31.7)	29.1 (19.9–38.2)	29.3 (20.2–38.3)	29.8 (24.4–35.1)	21.9 (16.6–27.1)	19.4 (16.0–22.8)
Barrier***	10.4 (6.5–14.3)	14.6 (6.3–23.0)	16.3 (8.7–23.8)	17.8 (10.0–25.6)	23.4 (14.4–32.5)	23.8 (18.7–28.8)	30.6 (24.8–36.5)	38.1 (33.9–42.2)
IUD***	21.5 (15.4–27.7)	14.8 (9.7–20.0)	13.4 (8.5–18.3)	11.9 (6.2–17.6)	11.6 (5.8–17.4)	9.4 (6.3–12.6)	7.8 (4.9–10.7)	7.2 (5.1–9.3)
Hormonal	8.5 (4.2–12.7)	10.9 (6.4–15.4)	13.8 (7.2–20.4)	9.3 (4.4–14.3)	8.1 (2.1–14.1)	13.0 (9.2–16.7)	13.0 (9.0–17.0)	11.1 (8.4–13.8)
Traditional***	34.7 (28.2–41.2)	33.6 (24.0–43.3)	27.9 (19.9–35.8)	31.3 (22.3–40.3)	24.2 (15.5–32.8)	22.7 (17.8–27.6)	24.8 (19.4–30.2)	23.6 (19.9–27.2)
Sterilization	0.0 (0.0–0.0)	0.0 (0.0–0.0)	3.3 (0.5–6.1)	0.0 (0.0–0.0)	1.3 (0.0–3.2)	1.0 (0.0–2.1)	1.3 (0.1–2.5)	0.6 (0.0–1.2)
**Other**	0.0 (0.0–0.0)	1.0 (0.0–2.4)	1.1 (0.0–2.6)	0.6 (0.0–1.7)	2.1 (0.0–5.0)	0.4 (0.0–1.1)	0.6 (0.0–1.4)	0.0 (0.0–0.0)
Other								
None	26.1 (23.5–28.6)	24.7 (21.8–27.6)	23.7 (21.1–26.3)	25.7 (23.3–28.1)	26.1 (23.6–28.5)	24.8 (22.5–27.1)	25.3 (23.1–27.4)	25.7 (24.1–27.4)
Barrier***	8.8 (7.1–10.4)	10.3 (8.3–12.4)	10.7 (8.7–12.6)	12.6 (10.7–14.4)	14.1 (12.1–16.1)	16.9 (14.9–19.0)	16.5 (14.6–18.4)	18.1 (16.6–19.5)
IUD***	35.9 (33.6–38.2)	35.9 (33.3–38.4)	34.3 (31.7–36.9)	30.3 (28.1–32.6)	26.9 (24.8–29.0)	23.6 (21.6–25.5)	24.0 (22.1–25.9)	22.5 (21.2–23.8)
Hormonal***	7.3 (5.7–9.0)	8.5 (6.5–10.5)	8.2 (6.6–9.7)	10.2 (8.4–11.9)	10.2 (8.5–11.9)	10.8 (9.2–12.5)	10.9 (9.4–12.5)	10.6 (9.5–11.7)
Traditional	20.7 (18.4–23.0)	19.8 (17.2–22.4)	22.1 (19.6–24.5)	19.6 (17.6–21.7)	19.9 (17.8–22.0)	21.1 (19.0–23.2)	21.2 (19.2–23.3)	20.7 (19.3–22.2)
Sterilization***	0.0 (0.0–0.0)	0.0 (0.0–0.0)	0.8 (0.4–1.2)	1.2 (0.6–1.9)	1.5 (1.0–2.1)	2.1 (1.5–2.8)	1.6 (1.0–2.2)	1.8 (1.4–2.2)
Other	1.3 (0.4–2.1)	0.8 (0.3–1.2)	0.4 (0.1–0.7)	0.4 (0.1–0.7)	1.3 (0.5–2.1)	0.6 (0.3–1.0)	0.5 (0.2–0.8)	0.6 (0.3–0.8)
Total	100.0	100.0	100.0	100.0	100.0	100.0	100.0	100.0

** p<.01.

*** p<.001.

*Notes:* P values refer to trends over time. Women were considered sexually active if they reported having had sex in the past 30 days. Women were excluded if they believed they were infertile or they were trying to conceive. Sterilization refers to either male or female sterilization. Percentages may not total 100.0 because of rounding. Numbers in parentheses are 95% confidence intervals.

While the prevalence of hormonal contraceptive use rose slightly among both the sample as a whole and respondents living in nonmetropolitan areas, it did not change significantly among residents of Moscow and St. Petersburg. However, the increase in prevalence of barrier method use was particularly large among residents of these cities (from 10% to 38%). Moreover, IUD use was less prevalent, and declined more steeply (from 22% to 7%), in these areas than elsewhere. In 1994, traditional methods were much more prevalent among metropolitan residents than among women in other regions (35% vs. 21%), but the prevalence of use of these methods fell more sharply in metropolitan areas, so that by 2003, the prevalence was similar in the two areas (24% and 21%, respectively).

**FIGURE 2:**
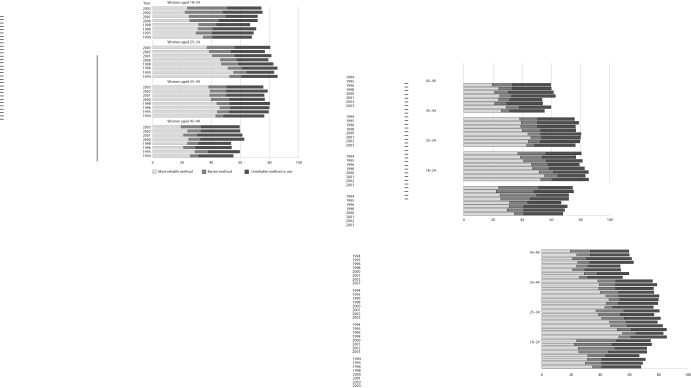
Percentage of sexually active women aged 18–49 who were using a contraceptive method, by reliability of method, according to age-group and year *Notes*: Women were considered sexually active if they reported having had sex in the past 30 days. Women were excluded if they believed they were infertile or they were trying to conceive. “Most reliable methods” are the IUD and hormonal contraceptives taken regularly; “barrier methods” denotes consistent use of condoms, diaphragms or spermicides; “unreliable methods” indicates use of other methods or inconsistent use of hormonal contraceptives or barrier methods.

The prevalence of reliable contraceptive use (IUD, consistently used hormonal contraceptives or consistently used barrier methods) increased slightly among 18–24-year-old women, but showed a small reduction among 25–34-year-olds (Figure 2). The difference was largely accounted for by an increase in the prevalence of barrier contraceptive use among the youngest women. However, the proportion of women using the most reliable contraceptives (IUD or hormonal contraceptives used regularly) declined in all age-groups, and particularly among women younger than 35.

The most common reason women did not use contraceptives was that they did not have sex regularly, reported by 26% of nonusers in 1994 and 29% in 2003 (Table 3, page 46). Other women did not use contraceptives because they wanted to get pregnant (the prevalence of this reason increased by more than two-thirds during the study, from 13% to 22%) or believed they were unable to conceive (which declined from 22% to 15%). The proportion of women who attributed their nonuse to finding contraceptives uncomfortable or unpleasant increased during the study period from 9% to 15%, but the prevalence of nonuse due to perceived health problems declined from 18% to 11%. In 1994, 9% of nonusers said they did not use contraceptives because they knew they could get an abortion; by 2003, the proportion had fallen to 6%. Expense was cited as a reason for nonuse more often in 1998 (the year of Russia's “ruble crisis”^26^) than in more recent years.

**TABLE 3:** Percentage distribution of sexually active women aged 18–49 who were not regularly using a contraceptive method, by reason for nonuse or irregular use, according to year

Reason	1994	1995	1996	1998	2000	2001	2002	2003
**Nonuse**	(N=1,355)	(N=1,258)	(N=1,065)	(N=1,500)	(N=1,629)	(N=1,762)	(N=1,857)	(N=3,732)
Does not have sex regularly*	25.8 (22.2–29.4)	29.3 (25.1–33.4)	30.4 (26.1–34.8)	30.0 (26.1–33.9)	31.6 (27.7–35.4)	32.6 (28.9–36.3)	29.6 (25.9–33.3)	28.8 (25.5–32.1)
Wants to get pregnant***	13.3 (10.3–16.2)	15.9 (12.4–19.4)	17.4 (13.7–21.1)	15.0 (11.9–18.1)	15.6 (12.6–18.6)	15.1 (12.4–17.9)	17.5 (14.5–20.4)	21.8 (18.7–24.9)
Is unable to get pregnant***	22.2 (19.1–25.3)	23.1 (19.6–26.6)	18.5 (15.5–21.5)	17.9 (15.1–20.8)	16.2 (13.2–19.1)	13.8 (11.4–16.2)	13.4 (11.2–15.7)	14.9 (12.4–17.5)
Feels contraceptives are uncomfortable or unpleasant***	9.3 (6.8–11.8)	8.9 (6.0–11.7)	6.4 (3.8–9.1)	7.6 (5.3–10.0)	9.0 (6.6–11.5)	15.9 (12.9–18.9)	16.2 (13.1–19.3)	15.0 (12.2–17.8)
Has health problems**	17.9 (14.9–20.9)	12.7 (10.0–15.4)	14.1 (11.1–17.2)	14.6 (11.8–17.5)	13.7 (11.1–16.3)	11.6 (9.2–14.1)	13.8 (11.3–16.4)	11.4 (9.2–13.7)
Could get abortion if necessary***	9.3 (6.9–11.8)	7.8 (5.4–10.3)	8.2 (5.7–10.7)	6.3 (4.3–8.3)	7.0 (4.7–9.2)	6.1 (4.1–8.1)	6.4 (4.4–8.4)	6.0 (4.2–7.8)
Cannot obtain a method	1.1 (0.2–2.1)	1.5 (0.2–2.8)	1.6 (0.0–3.2)	2.9 (1.4–4.5)	4.2 (2.4–5.9)	1.6 (0.7–2.5)	2.0 (1.1–2.9)	1.6 (0.7–2.5)
Cannot afford a method*	1.0 (0.2–1.8)	0.7 (0.0–1.5)	3.3 (1.3–5.2)	5.6 (3.6–7.6)	2.7 (1.5–3.9)	3.2 (1.7–4.7)	1.0 (0.3–1.7)	0.5 (0.0–1.2)
**Irregular use**	(N=158)	(N=103)	(N=112)	(N=129)	(N=121)	(N=128)	(N=149)	(N=244)
Does not have sex regularly	26.5 (18.1–34.9)	41.2 (29.0–53.4)	43.7 (34.2–53.1)	35.3 (26.4–44.2)	39.4 (29.6–49.2)	24.9 (17.5–32.2)	35.7 (27.3–44.1)	29.8 (19.3–40.2)
Wants to get pregnant	10.8 (5.1–16.5)	12.6 (4.4–20.9)	9.3 (3.1–15.5)	3.1 (0.0–6.9)	6.1 (2.1–10.1)	10.1 (3.6–16.7)	9.7 (4.8–14.6)	6.6 (2.3–10.9)
Is unable to get pregnant	2.3 (0.1–4.5)	4.0 (0.0–9.5)	0.0 (0.0–0.0)	4.6 (0.4–8.9)	4.5 (0.2–8.8)	5.6 (1.6–9.5)	4.1 (0.1–8.1)	0.0 (0.0–0.0)
Feels contraceptives are uncomfortable or unpleasant†	16.0 (9.5–22.6)	13.7 (7.3–20.1)	13.1 (6.0–20.2)	10.7 (5.1–16.3)	20.3 (11.7–28.9)	29.1 (19.8–38.3)	21.6 (14.6–28.6)	26.8 (15.8–37.7)
Has health problems	12.1 (6.6–17.7)	4.2 (0.3–8.2)	5.0 (0.3–9.6)	6.9 (2.6–11.2)	2.3 (0.0–5.1)	4.3 (0.5–8.1)	4.0 (0.5–7.5)	11.6 (4.1–19.1)
Could get abortion if necessary	18.4 (11.8–25.0)	17.4 (7.4–27.5)	17.4 (9.2–25.5)	18.3 (9.7–26.8)	16.4 (8.0–24.7)	9.0 (3.5–14.5)	10.0 (4.7–15.4)	14.5 (6.8–22.3)
Cannot obtain a method	11.2 (5.0–17.3)	4.0 (0.0–8.4)	9.0 (3.1–14.8)	10.2 (3.8–16.5)	7.1 (2.3–11.9)	10.7 (4.3–17.1)	12.4 (6.2–18.6)	10.2 (3.4–16.9)
Cannot afford a method	2.7 (0.1–5.2)	2.9 (0.0–6.8)	2.6 (0.0–6.6)	10.9 (3.6–18.3)	3.9 (0.0–8.9)	6.4 (1.4–11.4)	2.4 (0.0–5.2)	0.6 (0.0–1.6)
Total	100.0	100.0	100.0	100.0	100.0	100.0	100.0	100.0

* p<.05.

** p<.01.

*** p<.001.

† p<0.1

*Notes:* P values refer to trends over time. Women were considered sexually active if they reported having had sex in the past 30 days. Percentages may not total 100.0 because of rounding. Numbers in parentheses are 95% confiderce intervals.

The proportion of barrier contraceptive users who had used their method irregularly varied between 8% and 14% during the study period, and the proportion of oral contraceptives users who had done so ranged from 7% to 12% (not shown). Among women who did not always use their primary method, lack of regular sex was generally the most common reason for irregular use (Table 3). The proportion of irregular users who cited discomfort or unpleasantness as their reason increased from 16% in 1994 to 27% in 2003; this trend was marginally significant. Barrier contraceptives were the methods most commonly associated with irregular use due to discomfort (not shown).

The multivariate analyses showed relatively few changes in the characteristics associated with reliable contraceptive use between 1994 and 2003 (Table 4, page 47). At both time points, women with a secondary or higher education were more likely than other women to be using reliable contraceptives (odds ratios, 1.5–1.7), and smokers were less likely to be doing so than nonsmokers (0.6–0.7). In addition, the odds of use were lower among women aged 35 or older than among women aged 25–34 (0.3–0.7). Previous childbirth (2.6) and household wealth (1.1) predicted reliable contraceptive use in 1994, but no longer did so by 2003. In contrast, binge drinking became a predictor of nonuse in the later time period, although this association was largely explained by social, demographic and other variables.

**TABLE 4:** Odds ratios (and 95% confidence intervals) from logistic regression analyses assessing associations between selected characteristics and reliable contraceptive use in sexually active women aged 18–49, 1994 and 2003

Characteristic	1994		2003	
		
	Model 1	Model 2	Model 1	Model 2
**Age**				
18–24	0.51 (0.38–0.69)	0.73 (0.51–1.05)	0.87 (0.67–1.12)	0.95 (0.68–1.33)
25–34 (ref)	1.00	1.00	1.00	1.00
35–44	0.66 (0.52–0.83)	0.58 (0.45–0.75)	0.77 (0.63–0.95)	0.65 (0.52–0.82)
45–49	0.28 (0.19–0.42)	0.28 (0.19–0.43)	0.42 (0.25–0.71)	0.32 (0.18–0.55)
**Marital status**				
Married/cohabiting (ref)	1.00	1.00	1.00	1.00
Single	0.42 (0.27–0.65)	1.22 (0.63–2.35)	0.77 (0.55–1.06)	0.62 (0.36–1.08)
Divorced	0.92 (0.65–1.31)	1.02 (0.68–1.54)	0.95 (0.68–1.35)	1.14 (0.78–1.66)
Widowed	2.47 (0.89–6.85)	2.81 (1.02–7.78)	1.27 (0.44–3.66)	1.63 (0.53–5.07)
**Residence**				
Moscow/St. Petersburg	0.59 (0.41–0.85)	0.64 (0.42–0.97)	1.12 (0.87–1.44)	1.20 (0.90–1.62)
Other (ref)	1.00	1.00	1.00	1.00
**Education**				
<secondary (ref)	1.00	1.00	1.00	1.00
Secondary	1.75 (1.26–2.44)	1.73 (1.21–2.47)	1.61 (1.19–2.18)	1.70 (1.20–2.39)
≥tertiary	1.62 (1.17–2.25)	1.74 (1.22–2.47)	1.67 (1.24–2.26)	1.52 (1.09–2.14)
**Household wealth**				
Asset score	1.04 (0.96–1.12)	1.14 (1.03–1.26)	1.02 (0.94–1.10)	1.05 (0.95–1.17)
**Smoking**				
Nonsmoker (ref)	1.00	1.00	1.00	1.00
Smoker	0.65 (0.49–0.87)	0.70 (0.49–1.00)	0.56 (0.44–0.72)	0.59 (0.44–0.80)
**Alcohol consumption**				
Moderate (ref)	1.00	1.00	1.00	1.00
Binge	1.07 (0.78–1.47)	1.26 (0.88–1.81)	0.60 (0.43–0.85)	0.73 (0.49–1.09)
None	1.23 (0.98–1.54)	1.27 (1.00–1.61)	1.16 (0.93–1.45)	1.05 (0.82–1.35)
**Ever gave birth**				
No (ref)	1.00	1.00	1.00	1.00
Yes	2.77 (1.36–5.61)	2.62 (1.30–5.31)	1.13 (0.68–1.86)	0.84 (0.49–1.44)
**Had abortion in past year**				
No (ref)	1.00	1.00	1.00	1.00
Yes	0.81 (0.53–1.24)	0.86 (0.55–1.35)	0.81 (0.53–1.25)	1.00 (0.66–1.54)
**Wants another child**				
No (ref)	1.00	1.00	1.00	1.00
Yes	0.98 (0.73–1.30)	1.21 (0.90–1.63)	0.93 (0.73–1.18)	0.67 (0.53–0.87)

*Notes:* Women were considered sexually active if they reported having had sex in the past 30 days. Women were excluded if they believed they were infertile or they were trying to conceive. Model 1 controls for age and marital status; Model 2 controls for age, marital status, residence, education, household wealth, smoking, alcohol consumption and previous childbirth. Results are adjusted for clustering by survey site (including census district). ref=reference category.

## DISCUSSION

### Patterns of Contraceptive Use

We hypothesized that during the study period, the level of reliable contraceptive practice would increase and a more homogenous pattern of use would emerge. However, we believe that we have disproved these hypotheses. Although overall contraceptive prevalence remained steady, the use of effective methods declined. This was because of the substantial decline in the use of IUDs, the minimal increase in hormonal contraceptive use, the large rise in the use of barrier methods and the continuing popularity of traditional methods. Nonuse and irregular use were common. Although the elevated rates of reliable contraceptive use seen in 1993 among more educated women and the reduced rates observed among smokers persisted 11 years later, the higher rates among women who had borne children had disappeared by 2004, perhaps indicating that initiation of contraceptive use as part of postnatal care had become less important.

The decline in IUD use and the corresponding increase in the use of barrier contraceptives were most pronounced among younger women and those in metropolitan areas. Several possible reasons may explain these trends. First, clinician knowledge or practice may have improved. Among women younger than 25 and those with chlamydia, IUDs have been linked to pelvic infection and to infertility,^27^ which affects 10–25% of married couples in Russia.^28^ Barrier contraceptives are the only ones that are effective in preventing the transmission of STDs, the prevalence of which (particularly syphilis and HIV) has risen exponentially, especially in Moscow and St. Petersburg.^29^,^30^

Changing attitudes among women may be another important reason for the rise in barrier method use, especially in the principal cities, where awareness of the need for HIV prevention is greater. Russia and Ukraine account for more than 90% of people living with HIV in Europe.^31^ Mass HIV screening in 1995 was followed by several safer-sex campaigns between 1998 and 2003,^32^ the latter part of our study period.

However, the relationship between attitudes and behavior is complex. Despite their knowledge of risk factors, Russians have a low level of perceived vulnerability to AIDS, and they frequently stop using condoms in the early stages of a new partnership or have multiple partners.^33^,^34^ Moreover, in one survey, one-third of respondents said they believed that condoms do not protect against HIV.^35^ Perhaps these factors explain the low prevalence in Russia of dual protection (protection against STDs using condoms, and against pregnancy using another method), although regional variation in the implementation of HIV prevention programs may also be a factor.^36^

The proportion of barrier contraceptive users who reported not always using their method ranged from 8% to 14%. These figures are lower than those in a Russian study of high-risk STD patients, in which two-thirds used condoms less than half the time.^37^ The difference most likely reflects the lower risk nature of the RLMS population (although information on their sexual behavior is limited).

Hormonal contraceptive use did not increase among 18–24-year-olds during the study period, although it rose slightly among women in their middle reproductive years. Hormonal contraceptives protect against pregnancy more effectively than barrier methods alone, do not (unlike IUDs) increase a woman's risk of pelvic infection and can be used with barrier methods as part of effective dual protection.^1^ However, hormonal contraceptive use was much lower in this study than in many western European countries (e.g., the United Kingdom, France and Denmark), although rates were similar to those in several eastern European nations.^38^ Again, attitudes may be important, as the low levels of use may reflect persisting effects of Soviet propaganda. Research on maternal health care has shown that many ineffective or dangerous practices dating from the Soviet period remain in widespread use.^39^ Injectable contraceptives and implants were rarely used in that era, and Russian women's knowledge of these methods has been limited.^18^

Another factor may be price. Except during hospitalization, individuals must pay for all pharmaceuticals, including oral contraceptives. Furthermore, substantial markups along the supply chain, coupled with fee splitting by physicians and pharmacists, may make oral contraceptives more expensive than other methods.^40^

The use of traditional methods has remained steady, and the similarity in prevalence across age-groups that we observed is consistent with findings from another study.^41^ The prevalence of traditional methods was particularly high (approaching 35%) in the two main cities during the mid-1990s, consistent with a 1993 study from St. Petersburg.^13^ By 2003, however, the prevalence of these methods in metropolitan areas was similar to that in nonmetropolitan areas (around 20%), where prevalence remained unchanged. It is surprising that traditional practices were more common in these relatively cosmopolitan cities than elsewhere. Overall, traditional methods continue to be used much more widely in Russia than in western Europe.^38^ Given that knowledge of modern contraceptive methods was almost universal by the 1990s,^18^ it is likely that attitudes are important; whether Russian women still believe that traditional methods are effective, as they did in the 1980s,^14^ or consider them safer than modern methods is not certain.

Attitudes toward contraception could be culturally influenced. At the start of this study, contraceptive use in Russia mirrored two patterns seen in other former communist countries. The first is the relatively high prevalence of IUD use, typical of several former Soviet republics, including Ukraine, Tajikistan, Kyrgyzstan and Uzbekistan. The second feature, the frequent use of traditional methods, is seen in eastern European countries and Ukraine.^38^ Despite some changes, these patterns persisted in 2003.

### Reasons for Nonuse

Women cited a variety of reasons for not using contraceptives. The health concerns mentioned by many women may reflect to some degree the lasting impact of Soviet propaganda.^14^ The proportion citing cost as an obstacle was highest in 1998, the year of the ruble crisis,^26^ but otherwise affordability and accessibility were rarely a problem, mirroring results from a 1995 survey.^41^ (We were unable to ascertain, however, whether cost was an issue for specific methods, such as oral contraceptives.) Irregular sexual relations was often mentioned, which may reflect the increasing rates of divorce and relationship breakdown in posttransition Russia.^42^ The proportion of women who did not practice contraception because they could get an abortion if they became pregnant declined only slightly, underscoring the continuing importance of abortion in Russia. Irregular users were particularly likely to cite the availability of abortion as a reason for not using contraceptives, suggesting that future research on the reasons for unprotected intercourse should consider the context of the sexual encounter. By 2003, discomfort was mentioned by 15% of nonusers and 27% of irregular users, indicating that, as elsewhere, comfort is an important factor in contraceptive choice.^14^ The increase in this reason may reflect the rising prevalence of barrier contraceptives.

The proportion of women who were not using contraceptives because they wanted more children increased between 1994 and 2003. The failure of Russia's low fertility rate to increase during the study period^22^ is therefore counterintuitive, all the more so given that the reported use of effective contraceptives did not increase in the RLMS and the national abortion rate continued to decline.^3^ Despite the limitations of the data, our findings do not support the view that the low level of fertility was related to improved access to family planning.

Various reasons have been proposed for Russia's low fertility rate. Infertility is common;^28^ however, the proportion of RLMS respondents who cited infertility as the reason for not practicing contraception declined between 1994 and 2003 (though subjective reports are hard to interpret conclusively). Reduced frequency of intercourse, given heightened concerns about HIV, is a possible explanation, but it is not likely.^37^ Age at marriage began rising in Russia during the early 1990s after a long decline; this might provide a partial explanation, but the marked rise in cohabitation makes this hard to study.^42^ Changes in birthspacing after the pronatalist policies of the 1980s were abandoned are another possible explanation, but it seems implausible that the effects would have persisted for two decades. In addition, prior abortion and plans for further children were associated with contraceptive practice in this study. The explanation may lie in the profound social and economic changes that occurred during the dissolution of the Soviet Union. Household economic conditions influence decisions about abortion^43^ and family size;^13^ in this study, education was more strongly associated with reliable contraceptive use than was material wealth, suggesting that the relationship between socioeconomic conditions and fertility is not necessarily straightforward.

Overall, considerable further research into the complex relationships among fertility, abortion and socioeconomic conditions, as well as the role of women's attitudes, is required to understand and address Russia's population decline.

### Limitations

This study had several potential limitations. Women younger than 18 were excluded, age-groups were fairly broad and data about sexual behavior (e.g., having multiple partners) were not available. Some women were interviewed in the presence of a family member, which may have influenced their responses. In addition, data from each round of the survey were analyzed separately, and despite considerable overlap in the study population in different years, losses and additions to the study occurred. Overrepresentation of individuals who were wealthy, single, divorced, less educated or urban residents^24^ may have affected the findings. We excluded from our analyses women who reported being infertile, but this was a subjective assessment, and some of those women may have been able to conceive. Nevertheless, our data show marked trends in contraceptive use, which these limitations are unlikely to explain.

## Conclusions

More research is needed to explain the widespread nonuse of contraceptives, to understand why traditional methods remain popular and to elucidate why effective methods, particularly the pill, may not be acceptable. Structural factors may influence contraceptive practice, and weak government support for family planning programs^5^,^10^ may partly explain the lack of an increase in effective contraceptive use. However, even well-implemented family planning programs in Russia are not always effective.^44^ Research should take place across Russia, since our analysis shows that findings from Moscow and St. Petersburg, which dominate the literature, may not be nationally representative.

Finally, policies on family planning should be linked with those on HIV and AIDS. Russia's HIV control policy has focused on drug abuse and the sex industry, but heterosexual transmission is becoming an increasingly important means of infection.^30^ Therefore, finding effective ways to promote condom use and dual protection will be important. These services should also be integrated with antenatal care, which will help improve contraceptive access for new mothers (which is poor^45^) and prevent vertical transmission of HIV.

## Footnotes

* These findings were consistent despite differences in study populations: Two studies examined parents with two children; another focused on persons who had married for the first time; the fourth focused on women attending polyclinics; and the last one examined women attending polyclinics who had one or more children.

† The survey was not conducted in 1997 or 1999.
